# Pompe disease treatment with twice a week high dose alglucoside alfa in a patient with severe dilated cardiomyopathy

**DOI:** 10.1016/j.ymgmr.2018.05.002

**Published:** 2018-05-21

**Authors:** Jesa L. Landis, Holly Hyland, Steven J. Kindel, Ann Punnoose, Gabrielle C. Geddes

**Affiliations:** a Medical College of Wisconsin, Medical School, United States; b Herma Heart Institute, Children's Hospital of Wisconsin, United States; c Medical College of Wisconsin, Department of Pediatrics, United States

**Keywords:** Pompe disease, Alglucoside alfa, Cardiomyopathy, Enzyme replacement therapy, Infantile onset Pompe disease

## Abstract

There is limited information regarding ideal dosage of alglucoside alfa in patients with Infantile Onset Pompe Disease (IOPD). The U.S. Food and Drug Administration approved alglucoside alfa at dosing of 20 mg/kg every other week, but there are small studies and case reports suggesting that dosing higher than this leads to improved ventilator free survival and development without adverse events. We review the clinical course and short term clinical outcomes one year following late diagnosis of IOPD in a 3 month old who presented severely affected and was treated with 40 mg/kg twice a week for 21 infusions until six months of age then transitioned to 40 mg/kg/week. The patient responded well to 40 mg/kg twice a week treatment without adverse reactions and significant clinical improvement.

## 1 Introduction

Since enzyme replacement therapy (ERT) with alglucosidase alfa was approved by the U.S. Food and Drug Administration (FDA) in 2006, the outcome for children with Infantile Onset Pompe Disease (IOPD) (OMIM #232300) has changed dramatically and is constantly evolving. ERT has improved IOPD clinical outcomes by prolonging survival, increasing length of ventilator-free survival, improving cardiac function, and allowing patients to achieve previously unachievable motor milestones [1]. The FDA approved dose of alglucosidase alfa is 20 mg/kg every other week (eow). Even with FDA approved dosing, 50% of treated infants require ventilator support by age three years [1]. Limited research on small cohorts suggests that there are improved outcomes when ERT is administered with increased frequency at higher doses [[1], [2], [3]]. Multiple studies in both humans and mice support potential benefits and safety of higher dosing [1,[3], [4], [5], [6], [7], [8]]. The data suggests that increasing the dose of ERT decreases the respiratory, cardiovascular, and motor decline [1,3]. A higher initial dose of ERT has been shown to yield better outcomes [1,3]. Dosing remains controversial with many centers varying dosing from between 20 mg/kg/eow up to 40 mg/kg/week. At our center all patients with IOPD are treated with 40 mg/kg/week.

## 2 Case summary

### 2.1 Clinical presentation and initial hospital course

Patient was a 3 month, 3 week old male born at 35.2 weeks gestation with a past medical history significant for hypotonia, poor feeding and failure to thrive attributed to his prematurity. Prior to admission, he presented to his primary care physician for congestion, cough and rhinorrhea. This was the third presentation to the primary care doctor for these symptoms, which were attributed to recurrent upper respiratory infections. A chest radiograph was obtained at this time demonstrating an enlarged cardiac silhouette. An echocardiogram demonstrated ejection fraction of 20% and the patient was transferred to Children's Hospital of Wisconsin (CHW) for further care.

Upon arrival to CHW, he was admitted to the cardiac intensive care unit and began treatment with diuretics (Aldactazide and Furosemide) and an Angiotensin Converting Enzyme (ACE) inhibitor (Enalapril). The suspicion for IOPD was high based on echocardiographic appearance and clinical examination. Alpha glucosidase enzyme activity was found to be low, consistent with a diagnosis of IOPD. The patient's heart failure worsened and milrinone was started on day six of hospitalization. ERT was also initiated on day seven of his hospitalization at 40 mg/kg/twice a week. ERT infusions were pretreated with 15 mg/kg Acetaminophen PO and 1 mg/kg Diphenhydramine IV due to concern that he did not have enough cardiac reserve to tolerate an infusion reaction.

At this time, the Cross Reactive Immunological Material (CRIM) status was unknown and 0.4 mg/kg Methotrexate was given subcutaneously daily for three days starting immediately prior to the first ERT infusion to prevent antibody formation. Mild thrush and lymphopenia were noted and Leucovorin rescue was initiated on day 5 of treatment. Another dose of 0.4 mg/kg methotrexate was administered subcutaneously with the third ERT infusion (day 7 of treatment). Milrinone was discontinued on day 9 of treatment. He was found to be CRIM positive by genotype (c.525delT (p.Gly176Argfs*45)/c.2481 + 102_2646 + 31del (p.gly828_Asn882del)) on day 11 of treatment. He was started on a beta blocker (Carvedilol) on day 12 of treatment in addition to an ACE inhibitor (Enalapril) and diuretics (Aldactazide and furosemide after ERT). Leucovorin was discontinued on day 12 of treatment.

**Fig. 1:**
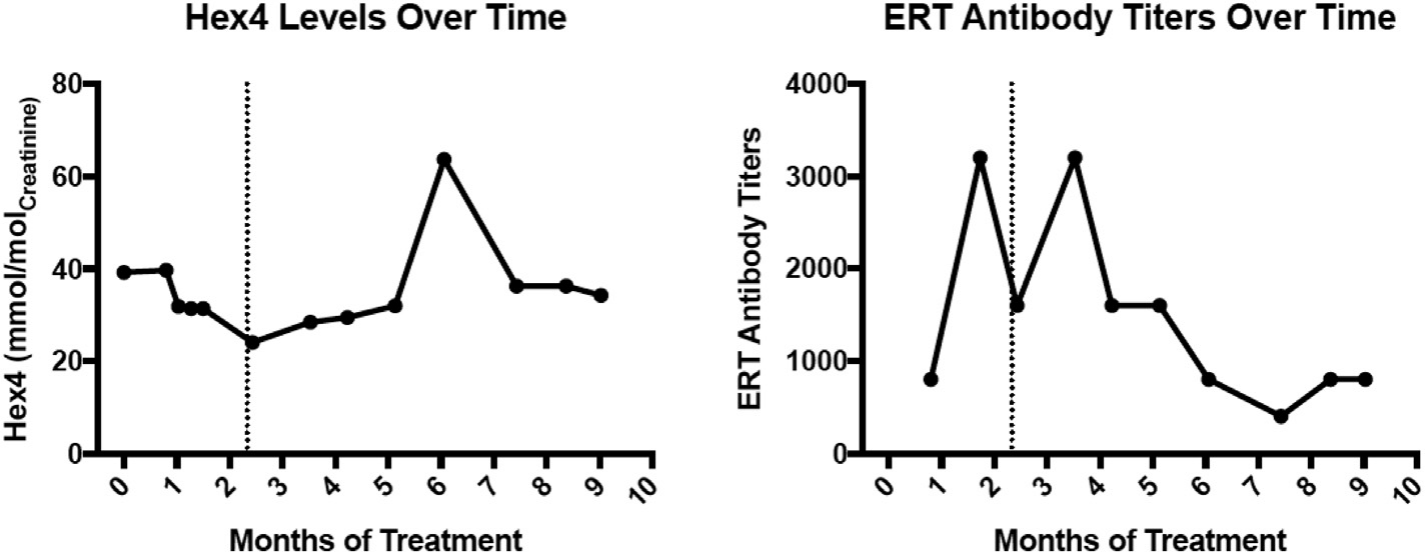
Hex4 (left) to monitor disease progression and ERT antibody to monitor immune response to ERT (right) levels over time with the dotted line denoting when the patient transitioned from 40 mg/kg alglucosidase alfa twice a week to 40 mg/kg alglucosidase alfa once a week.

The patient was transferred from the cardiac intensive care unit to a lower acuity medical unit on day 19 of treatment. He continued to progress well and the remainder of his hospitalization was uneventful. He never required respiratory support or supplemental oxygen. He was discharged day 32 of treatment. Urine Hex4 and ERT antibody levels were monitored monthly and are demonstrated in Fig. 1 [9]. He was found to have silent aspiration and was reliant on nasogastric tube feeding until a gastrostomy tube was placed. The patient underwent intravenous catheter placement and gastrostomy tube placement at eight months of age.

### 2.2 Cardiac course

**Fig. 2:**
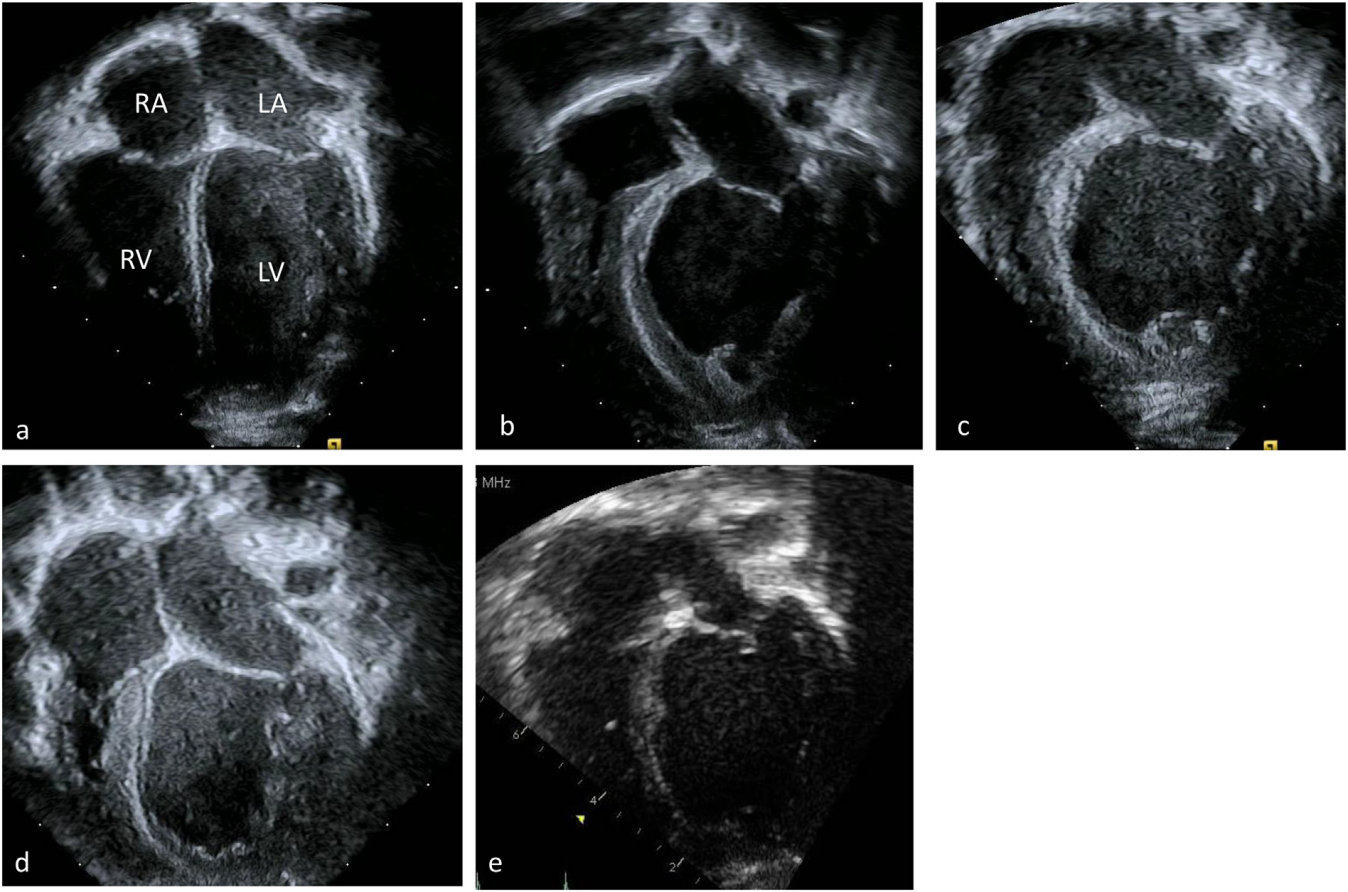
Four chamber apical echocardiographic view of a (a) normal heart with a similar view of this patient's hearts at (b) time of diagnosis (c) two months after initiation of ERT (d) four months after initiation and (e) a year after initiation. These images emphasize the dilation of this patient's left ventricle. RA right atrium, LA left atrium, RV right ventricle, LV left ventricle.

**Fig. 3:**
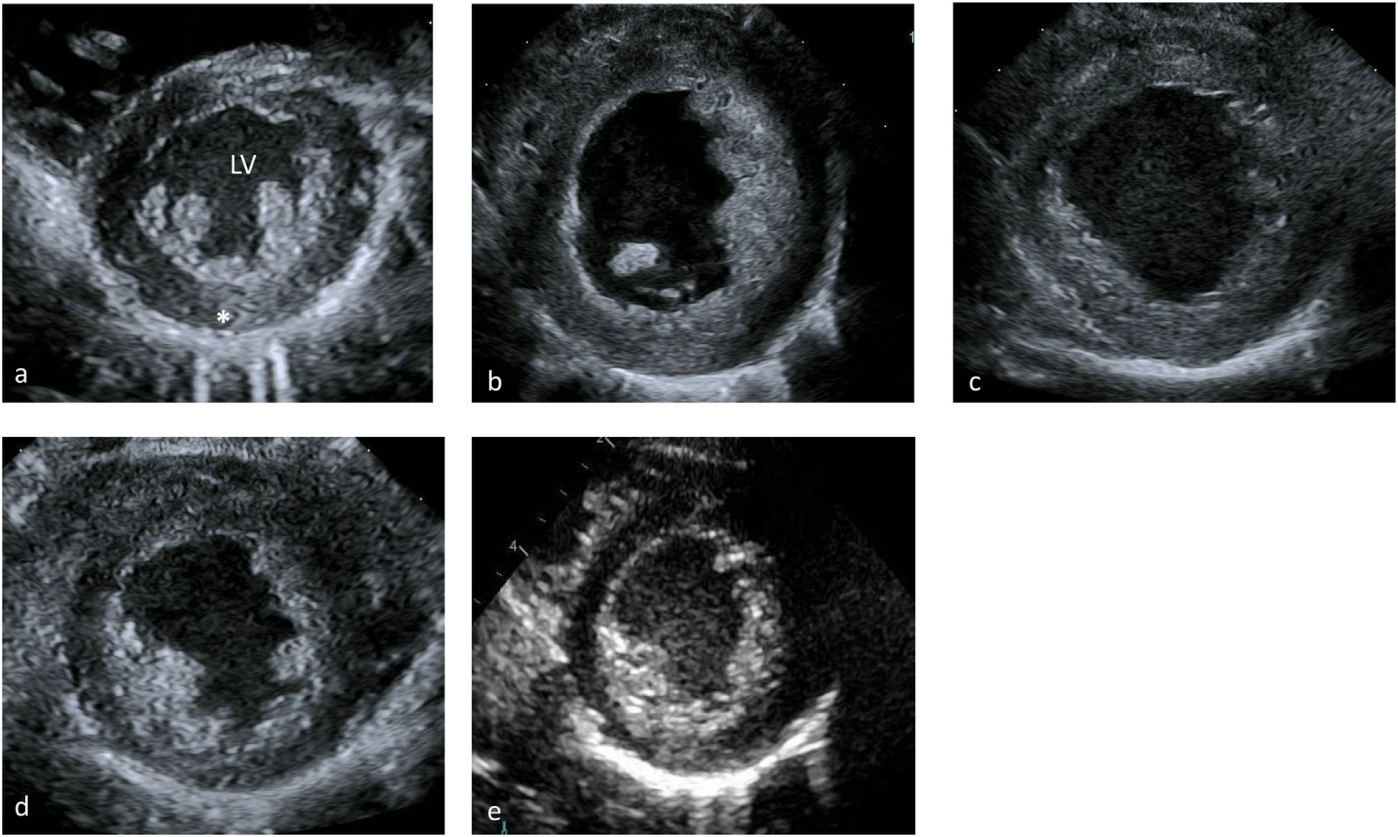
Parasternal short axis echocardiographic view of a (a) normal heart with a similar view of this patient's hearts at (b) time of diagnosis (c) two months after initiation of ERT (d) four months after initiation and (e) a year after initiation. These images emphasize the thickness of the left ventricular wall and improvement during the course of treatment. LV left ventricle, asterisk is on the left lateral free wall of the left ventricle.

The changes in the patient's echocardiograms over time of treatment are illustrated in Fig. 2 and Fig. 3. At presentation he had a severely dilated left ventricle (Fig. 2B) with mild to moderate hypertrophy (Fig. 3B). Left ventricular mass index (LVMI) was 251 g/ht^2.7^. There was severely diminished left ventricular systolic function with an ejection fraction of 20% (Normal 55–70%). While right ventricular systolic function was normal, it was impinged upon by the left ventricular dilation. NT Pro Brain Natriuretic Peptide (NT Pro-BNP) at presentation was 19,800 pg/mL. After 16 ERT infusions (54 days of treatment), the echocardiogram demonstrated LVMI was 165 g/ht. ^2.7^. There was similar levels of left ventricular dilation (Fig. 2C) and a mild decrease in left ventricular hypertrophy (Fig. 3C). NT Pro-BNP after 16 ERT infusions (57 days of treatment) was 2240 pg/mL. After four months of treatment LVMI was 121 g/ht^2.7^, left ventricular ejection fraction was 26%, and NT Pro-BNP was 207 pg/mL. The improvement in left ventricular dilatation is visible (Fig. 2D) and there is continued improvement of left ventricular hypertrophy (Fig. 3D). After one year and two weeks of treatment LVMI was 64 g/ht^2.7^ and left ventricular ejection fraction had improved up to 47% (normal 55–70%). The left ventricular dilation has significantly improved (Fig. 2E) and the left ventricular hypertrophy is not appreciable (Fig. 3E).

### 2.3 Developmental course

At time of initiation of ERT patient had very little movement, no head control, was unable to move his limbs against gravity, and unable to make facial expressions to the point he was unable to keep his eyes closed while sleeping. The day after his first ERT infusion minimal facial expression was noted. Patient had a physical therapy evaluation after two infusions (5 days of treatment). In supine, he was unable to bring his hands together, bring his hands to his face, or move his low extremities against gravity. In prone, he was unable to lift or turn his head. In supported upright sitting, he demonstrated no head control. Patient was able to intermittently activate neck flexors and extensors in supported upright sitting, rotate head 45 degrees towards R and L in supine, flex shoulders to 25 degrees in supine, and demonstrated active palmar and plantar grasp. Following 3 infusions (8 days), patient had an occupational therapy evaluation. Patient able to actively grasp toy placed in hand, and actively flex shoulders to 45 degrees and maintain this position for 3 s in reclined sitting. Following five infusions (15 days), in supine, patient was able to actively flex and extend bilateral knees and hips, actively dorsiflex and plantarflex bilateral ankles, and bring bilateral hands to mouth. Patient also demonstrated active thumb opposition with grasp. After seven infusions (22 days), patient was able to lift and turn head when being held upright at caregiver's chest, and demonstrated active chin tuck in supported upright sitting. After eight ERT infusions (26 days), he demonstrated midline head control for up to 2 s in supported upright sitting, was able to turn his head from left to/from right in prone on surface, was able to hold a lightweight rattle for 2–3 s, and was able to maintain hands to mouth for 6–8 s. After nine ERT infusions (29 days), he was able to actively flex his lower trunk off surface in supine and maintain UE reach for 10 s in supine. After 10 ERT infusions (33 days) he was able to maintain midline head control for up to 3 s in supported upright sitting and was able to actively adduct hips against gravity in supine. There was a one week gap between infusion 10 and 11 due to a holiday. After 13 ERT infusions (47 days), patient was able to reach right hand to right upper leg in supine, and roll from supine to right sidelyinig independently. After 14 ERT infusions (47 days), patient was able to transfer toys between hands and began to inconsistently activate lateral neck muscles for lateral head righting. After 21 ERT infusions (75 days) he demonstrated “bobbing” head control for two minutes and five seconds. After 21 ERT infusions (75 days) he was taken to 40 mg/kg/weekly infusions. He continued to progress developmentally. He currently (16 months of age) can roll, sit independently, and propel self in an infant wheelchair.

## 3 Discussion

Reports by Chen Lei-Ru et al. demonstrate that systolic dysfunction in IOPD usually occurs after 5 months of age [10]. They also note that cardiac remodeling can be unpredictable after 5 months of age particularly in the presence of systolic dysfunction and these patients seem to have a less favorable prognosis. They speculate this is due to a loss of myofibrils caused by accumulation of glycogen in the cytosol resulting in systolic dysfunction [10]. There was concern that the driver of the poor response was the process creating the systolic dysfunction as compared to the age as reported in the study [10]. Given the severity of systolic dysfunction noted in our patient at only 3 months of age we treated aggressively with a dose of 40 mg/kg/twice weekly to try to promote maximal cardiac remodeling to occur.

In addition to significant cardiac dysfunction the patient had severe hypotonia, and while at time of presentation he had adequate respiratory function it was not clear if respiratory dysfunction would evolve. A study comparing 20 mg/kg/eow to 40 mg/kg/week found that patients on 40 mg/kg/week had fewer respiratory infections and hospitalizations, and that patients whose dose was increased had decreases in respiratory infection following dose increase [1]. This study also found suggestion that higher dosing may be more beneficial in patients with more severe IOPD [1]. Studies looking at the level of alpha glucosidase activity within skeletal muscle also support using higher doses of alglucosidase alfa suggesting skeletal muscle activity only normalized with 40 mg/kg/week dosing [7,8,11]. Given this patient's level of decompensation and cardiac dysfunction, preservation of respiratory function and reduction of infection risk was critical until his cardiac function improved to a more stable level and contributed to the rationale of a dose of 40 mg/kg/kg twice a week.

When therapy was initiated the initial plan had been to give 40 mg/kg twice a week for two weeks and then decrease the dose to weekly. The duration of 40 mg/kg twice a week treatment was extended based on the significant response and significant improvements the patient had in a rapid timeframe.

We speculate that this increased dose may have decreased the length of stay the patient required in the cardiac intensive care unit (19 days after starting treatment) and his total hospital length of stay (discharged 32 days after starting treatment). There were no adverse events in our patient, it should be noted that the drug was maximally concentrated and diuretic dosing was given after the infusion to minimize risk for volume overload. We suggest that administering 40 mg/kg twice a week alglucosidase alfa in severely affected and decompensated patients with IOPD is a reasonable consideration to aid in recovery. Our patient demonstrated increased levels of Hex4 following transition to 40 mg/kg/week and it is possible that 40 mg/kg twice a week suggesting twice a week dosing kept him under better control. While this was true for our patient there is substantial clinical variation among IOPD patients and there have been patients with documented cardiac improvement on lower dosing [10].
